# The Strength and Delamination of Graphene/Cu Composites with Different Cu Thicknesses

**DOI:** 10.3390/ma14112983

**Published:** 2021-05-31

**Authors:** Song-Mi Kim, Woo-Rim Park, Oh-Heon Kwon

**Affiliations:** 1 Department of Safety Engineering, Graduate School, Pukyong National University, Busan 48513, Korea; miyaong2527@pukyong.ac.kr; 2 Department of Safety Engineering, Pukyong National University, Busan 48513, Korea; eeekg53@naver.com

**Keywords:** graphene/Cu composites, molecular dynamics, RVE, delamination

## Abstract

This study analyzed the mechanical and fracture behavior of graphene/copper (Cu) composites with different Cu thicknesses by using molecular dynamics (MD) and representative volume element (RVE) analysis. Three graphene/Cu composite analytical models were classified as 4.8, 9.8, and 14.3 nm according to Cu thicknesses. Using MD analysis, zigzag-, armchair-, and z (thickness)-direction tensile analyses were performed for each model to analyze the effect of Cu thickness variation on graphene/Cu composite strength and delamination fracture. In the RVE analysis, the mechanical characteristics of the interface between graphene and Cu were evaluated by setting the volume fraction to 1.39, 2.04, and 4.16% of the graphene/Cu composite model, classified according to the Cu thickness. From their obtained results, whether the graphene bond is maintained has the greatest effect on the strength of graphene/Cu composites, regardless of the Cu thickness. Additionally, graphene/Cu composites are more vulnerable to armchair direction tensile forces with fracture strengths of 14.7, 8.9, and 8.2 GPa depending on the Cu thickness. The results of this study will contribute to the development of guidelines and performance evaluation standards for graphene/Cu composites.

## 1. Introduction

Graphene, a structure with a hexagonal arrangement of carbon (C) atoms, is a thin and light nanomaterial with a thickness of 0.14 nm and a density of 0.77 mg/m^2^. Graphene is highly flexible and does not lose its intrinsic electrical conductivity when its area increases by more than 10% or it is bent. In addition, graphene has a 0.5–1 TPa elastic modulus [1,2,3]. As such, graphene has infinite potential as a composite material due to its outstanding physical properties.

To produce graphene on an industrial scale, it is necessary to consider a large area, high quality, and cost effectiveness. For instance, Zhuo et al. [4] produced graphene by using an insulating substrate without a transfer process. Kamedulski et al. [5] made graphene from graphite through wet chemical exfoliation methods.

Nevertheless, mass-producing graphene for commercialization is still difficult. The acquisition of high-quality large-area graphene for commercialization inevitably poses the risk of defects in the manufacturing process. To overcome this limitation, previous studies have continued the efforts to utilize graphene and metals as composite materials (composites). Since graphene has a large specific surface area and is composed of small atoms, using it in graphene/metal composites can facilitate graphene manufacturing and enhance existing metal materials with small volumes, which satisfies the strength and weight requirements and improves important mechanical properties such as tensile strength and elastic modulus [6,7].

In related studies, Kim et al. [8] revealed the strength effect associated with single-atomic layer graphene in graphene/metal nanocomposites, and Boostani et al. [9] combined graphene with aluminum (Al) to achieve high tensile strength and ductility. Additionally, Tang et al. [10] improved the mechanical properties of graphene/copper (Cu) composites by using graphene and nickel (Ni) composites, and Rezaei [11] identified deformation mechanisms in graphene/metal nanolayered composites by determining their tensile mechanical properties.

In particular, efforts are being made to composite Cu, which is widely used in semiconductor wiring and electronic devices, with graphene. Cu is a material with excellent electrical properties, and when used in combination with graphene, it is possible to add the excellent properties of graphene to the properties of Cu.

In other related studies, Kim et al. [12] developed graphene/Cu shell nanowires as high-performance electric cables with maximized mechanical toughness by contacting graphene and Cu at the interface and improving the electrical and thermal properties. Moreover, Hwang et al. [13] improved the mechanical properties of graphene/Cu nanocomposites with molecular-level processes, and Peng et al. [14] investigated the reinforcement mechanism of graphene/Cu composites by using a molecular dynamics (MD) approach.

Nevertheless, previous studies have focused on the strength improvement effect or electrical/thermal efficiency problems in graphene and Cu composites, and the usage specifications and performance of graphene/Cu composites for mass production or commercialization have not been fully researched. Furthermore, fracture characteristics such as interface delamination, which inevitably occurs when combining graphene and Cu, have not been elaborated on.

Therefore, according to these needs, this study evaluated the mechanical properties and delamination characteristics of graphene/Cu composite fractures according to two analytical methods. First, since the excellent properties of graphene result from its atomic structure, the MD approach, which is a suitable method for calculating intrinsic fracture phenomena that considers the atomic bonds of materials, was performed to evaluate the characteristics of the graphene/Cu composite interface.

Subsequently, representative volume element (RVE) analysis was performed to evaluate the mechanical properties associated with the interface between graphene and Cu, which extended from the microscopic dimension to the macroscopic dimension. RVE analysis is a technique that evaluates the properties of composites at a microscopic scale. This technique is independent of MD analysis and is extended to the microscopic scale, which is the scale at which graphene/Cu composites are used. The derivation of material properties at the macroscopic scale and the close examination of the characteristics of the interface between materials are possible by performing a distribution shape analysis of the mixed constituents in composites. As such, this study will be useful in the development of guidelines and performance evaluation criteria for graphene/Cu composites, because it is performed as an evaluation of graphene/Cu composites according to Cu thickness by controlling the thickness of Cu that is easy to manufacture.

## 2. Methodology and Modeling

### 2.1. Methodology

#### 2.1.1. MD Analysis

Carbon atoms in graphene arrange in either zigzag or armchair configurations. This means that even if the same tensile force is applied to graphene, crack propagation may vary depending on the direction of the tensile force. Therefore, MD is required to estimate the effects of the graphene atomic arrangement.

The MD technique is known to be the most suitable method for computing changes in the dynamic properties of nanomaterials. Nanomaterials such as graphene have large specific surface areas; therefore, the phenomena that occur at interfaces have a significant impact on their characteristics. An MD analysis allows us to account for the structural features associated with the atomic arrangement of graphene, calculate the interactions between the graphene and Cu molecules as tensile forces are loaded, analyze the mechanical behaviors, and obtain the strength and strain values. In this study, the large-scale atomic/molecular massively parallel simulator (LAMMPS) [15] was used as an analytical simulation tool to implement MD simulations and is specialized for modeling and computing the mechanical properties of materials.

In general, LAMMPS analysis proceeds in three stages. The first step involves the creation of an initial model that consists of the number of atoms that shapes the analytical model. To produce composites, each of the two materials must be modeled before the composites are modeled. In this paper, after modeling graphene and Cu separately, two models were used for graphene/Cu composites, and three new models were produced to evaluate different Cu thicknesses. The Cu thickness was modeled as 5, 10, or 15 nm in the graphene/Cu composites in accordance with previous studies in the semiconductor field that used <10 nm graphene in processing [16,17].

Moreover, the potential of each atom was determined, as this is the most appropriate method to calculate the interactions between atoms. In this study, Tersoff [18] potentials, which are specialized for calculating the interatomic interactions of covalent bonds in materials, were used for graphene, in which carbon atoms bond covalently. Cu was calculated by using the EAM/FS [19] potential, an advanced version of the conventional EAM potential developed by Finnis and Sinclair that accurately calculates intermolecular interactions in metals and metal alloys and complements the accuracy of force calculations used to simulate pores or cracks in metals and the cohesive energy of a surface or interface.

The second step involves an equilibrium stage, in which Newtonian equations are used to interpret the process (equilibrium phase) in which the position and speed of the atoms that make up the molecules are independent of changes over time. It is necessary to obtain only the desired properties, with the synthesized interatomic bonds acting as compounds. The concept of a computer simulating the interaction between atoms is called an ensemble, and ensembles should be determined in the equilibrium phase.

The last step involves calculating the physical quantities. Equilibrium graphene/Cu composite models are subjected to tensile analysis to evaluate their failure and strength under various conditions. A tensile analysis should define the tensile direction and velocity. Specific inputs and interpretation conditions for each step are described in Table 1 of Section 3.

#### 2.1.2. RVE Analysis

The physical properties of composites depend on the size, volume fraction, shape and mixed structure of the component materials; thus, an evaluation of the RVE, which models microcomposites, is important [20]. The RVE is a unit cell that can represent the microstructure of mixed composites. In this study, the ABAQUS plug-in program, micromechanics, was used to generate the RVE model, and this model was homogenized with periodic boundary conditions (PBCs) that were allowed to infinitely expand.

PBCs are a concept used in both RVE analysis and MD. If the simulation box is periodic, then atoms interact across the boundary, and they can exit from one end of the box and enter in the other end again. As a result, since the total number of molecules in the box remains constant, it can be homogenized, as the meaning of the boundary disappears within the system as a whole.

PBCs are applied evenly according to the characteristics and shape of the composites. Specifically, for each interface, the boundary conditions are applied by binding the top and bottom surfaces and the left and right surfaces. This is the governing equation for the PBCs in Equation (1).

(1) $$\Phi \left(x_{j}+p_{j}^{\alpha }\right)=\;\Phi \left(x_{j}\right)+\left(\frac{\partial \Phi }{\partial x_{j}}\right)\;p_{j}^{\alpha }$$

In Equation (1), $x_{j}$ represents an arbitrary coordinate point, and $p_{j}^{\alpha }$ represents a vector in the$\;\alpha^{th}$ direction. The plug-in program applies Equation (1) to the RVE boundary node as a constraint so that the model has periodicity. When the periodic condition is satisfied, the analysis is performed to calculate the macroscopic-scale tensor component$\;\bar{C}$.

(2) $$\bar{\sigma }=\bar{C}:\bar{\varepsilon }$$

The governing equation is Equation (2). $\bar{\varepsilon }$ and $\bar{\sigma }$ represent the strain tensor and stress tensor, respectively, at the macroscopic scale. Equation (3) calculates the tensor component of column 1 by setting the value of $\varepsilon_{11}$ equal to 1 and all remaining columns equal to 0 in Equation (2). When $\varepsilon_{11}$ is equal to 1, the macroscopic-scale stress ($\bar{\sigma }$) is calculated as the mean stress in a volume (V) within the RVE model through Equation (4) [21].

(3) $$\left[\begin{array}{l} \sigma_{11}\\ \sigma_{22}\\ \sigma_{33}\\ \sigma_{12}\\ \sigma_{13}\\ \sigma_{23} \end{array}\right]=\left[\begin{array}{l} D_{1111}\;D_{1122}\;D_{1133}\;D_{1112}\;D_{1113}\;D_{1123}\\ D_{2211}\;D_{2222}\;D_{2233}\;D_{2212}\;D_{2213}\;D_{2223}\\ D_{3311}\;D_{3322}\;D_{3333}\;D_{3312}\;D_{3313}\;D_{3323}\\ D_{1211}\;D_{1222}\;D_{1233}\;D_{1212}\;D_{1213}\;D_{1223}\\ D_{1311}\;D_{1322}\;D_{1333}\;D_{1312}\;D_{1313}\;D_{1323}\\ D_{2311}\;D_{2322}\;D_{2333}\;D_{2312}\;D_{2313}\;D_{2323} \end{array}\right]\left[\begin{array}{l} 1\\ 0\\ 0\\ 0\\ 0\\ 0 \end{array}\right]\Rightarrow \left[\begin{array}{l} D_{1111}\\ D_{2211}\\ D_{3311}\\ D_{1211}\\ D_{1311}\\ D_{2311} \end{array}\right]$$

(4) $$\bar{\sigma }=\frac{1}{V}\;\overset{}{\underset{V}{\int }}\sigma \left(x\right)dV$$

### 2.2. Modeling

#### 2.2.1. MD Modeling

**Figure 1:**
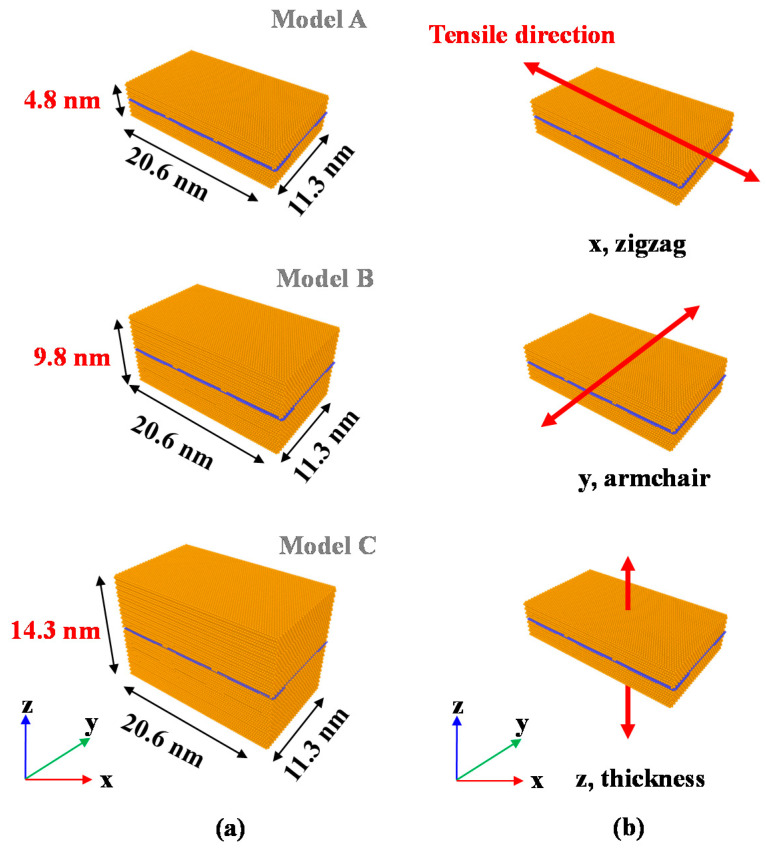
MD model for graphene/Cu composite modeling conditions: (**a**) produced model sizes; (**b**) formed tensile directions of zigzag-, armchair-, and z (thickness)-directions.

In the modeling phase, the analytical model is divided into three separate models according to the thickness of Cu, as shown in Figure 1.

The thicknesses used in models A, B and C were 4.8, 9.8 and 14.3 nm, respectively. The thickness of Cu was determined by referring to studies from the semiconductor field in which the product thickness was less than 10 nm when graphene was used. Subsequently, the specific condition of the atomic system in the equilibrium phase, the ensemble, was used to produce an NVT ensemble. NVT ensembles are suitable ensembles that can be used in tensile analysis because they reduce the incidence of errors in mechanical property predictions by considering the number of atoms (N), volume (V), and temperature (T). In the tensile analysis step for each axis, the boundary condition was set to 0.6 nm at both ends of the axis. For graphene, there were three types of tensile forces applied to each axis (x, y, and z), and the tensile speed was 0.017 nm/ps. The x-axis is the zigzag direction, and the y-axis is the armchair direction. The z-axis is in the direction of the thickness of the structure. The analytical cases were distinguished based on the load direction. Cracks exist in the zigzag and armchair directions which are feature of hexagonal graphene. Under these conditions, the characteristics of both the armchair direction and the zigzag direction of graphene were determined. The following were used to describe the conditions under which bonds were broken: when the stress approached zero; when the hexagonal arrangement inherent to graphene was broken; and when Cu was completely fractured.

#### 2.2.2. RVE Modeling

**Figure 2:**
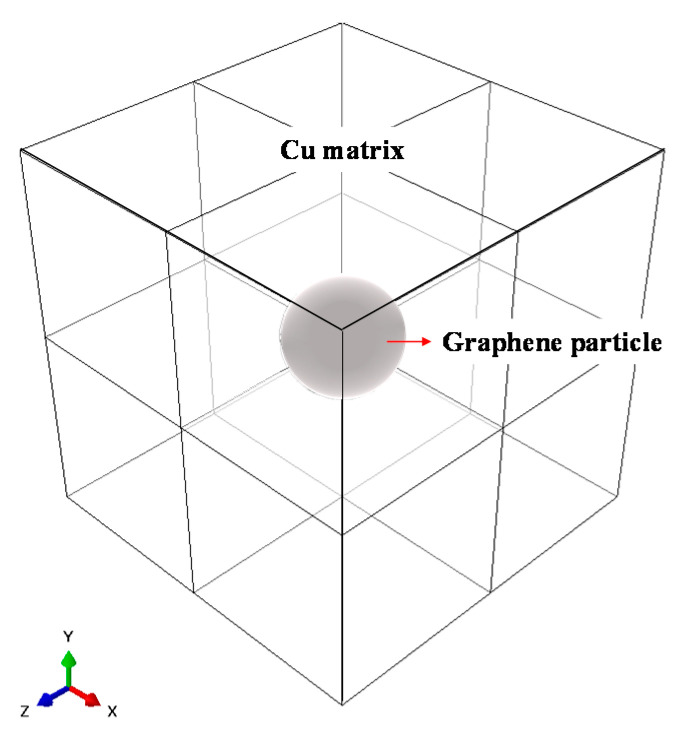
RVE model for graphene/Cu composites.

The RVE analytical model is composed of Cu and graphene, as illustrated in Figure 2.

The RVE model comprises three separate models, and the volume fraction of graphene in each model was specified as 1.39, 2.04, or 4.16%. These criteria result from calculating the volume fraction of graphene and Cu in the three models by using MD. The properties of graphene and Cu are shown in Table 1.

**Figure 3:**
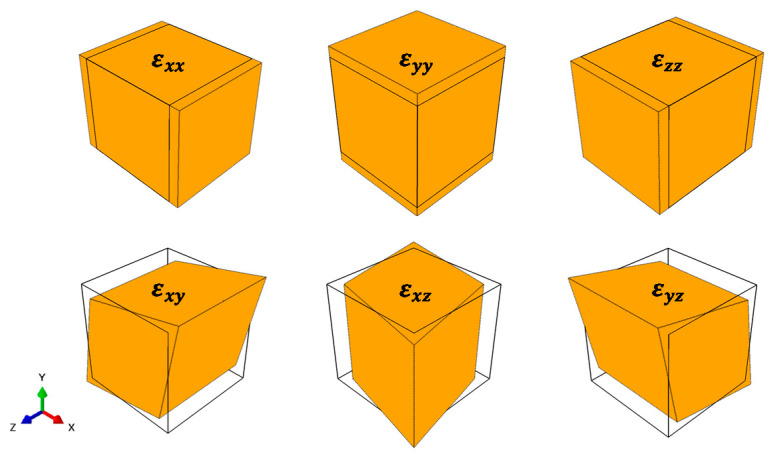
Schematic diagram of a finite element model for RVE perturbation analysis.

The 1.39% value results from calculating the volume fraction of model A with MD by using the largest Cu thickness, where the 2.04% value is the volume fraction of model B calculated with MD, and the 4.16% value is the volume fraction of model C calculated with MD by using the smallest Cu thickness. The boundary condition for each RVE analytical model includes six analytical cases in which the center point of the RVE model is fixed (Ux = Uy = Uz = 0), and the strain in each direction ($\varepsilon_{11},\;\varepsilon_{22},{\;\varepsilon }_{33},{\;\varepsilon }_{12},{\;\varepsilon }_{13},{\;\varepsilon }_{23}$) is set to 1, as shown in Figure 3.

Perturbation analyses that do not affect one another are applied to each analytical case to satisfy the periodicity. In the RVE analysis, 137,115 nodes were assigned, and a total of 133,512 eight-node linear brick elements were used.

## 3. Results and Discussion

### 3.1. Results of the MD Analysis

#### 3.1.1. Results of the Zigzag-Direction Tensile Analysis

**Figure 4:**
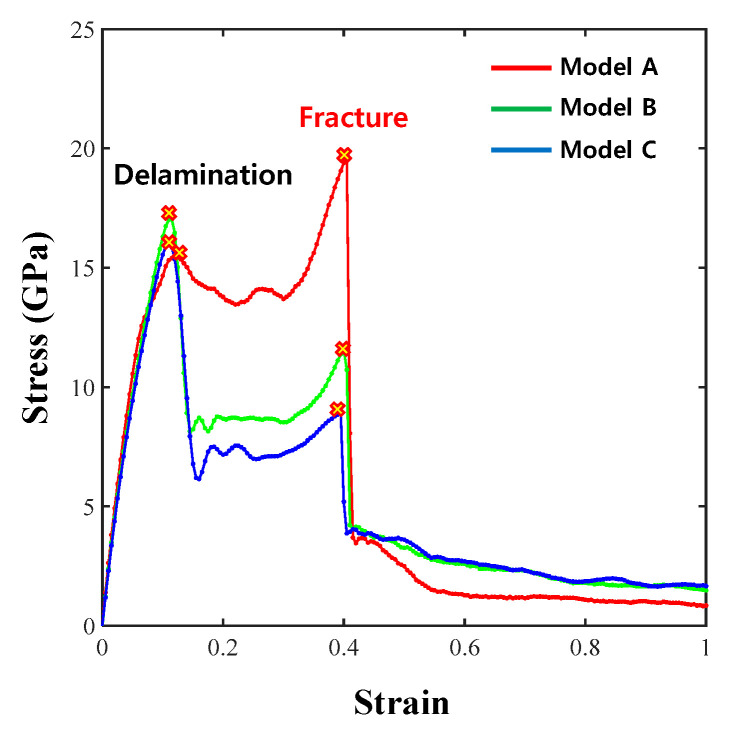
Zigzag direction σ–ε results for models A, B, and C.

The results of the analytical model applied with the tensile force in the zigzag direction are shown in Figure 4.

The first delamination event occurred when the strain reached approximately 0.1 in models A, B and C, and the strain at the time of fracture was 0.4. Model A had the lowest stress value among the three models at the time of delamination, with values of 15.56 GPa for model A, 17.02 GPa for model B and 16.03 GPa for model C. When the structure completely fractured, the fracture stresses of models A, B, and C were 19.7, 11.5 and 8.9 GPa, respectively, and the fracture strength value decreased as the thickness increased. The overall fracture behavior of models A, B, and C showed the fracture of the entire graphene/Cu composite after interfacial delamination occurred. The fracture stress of model B was lower than that of model A. These results suggest that the graphene bond affects the overall graphene/Cu composites and that the thickness of Cu does not improve the strength of the overall graphene/Cu composites.

**Figure 5:**
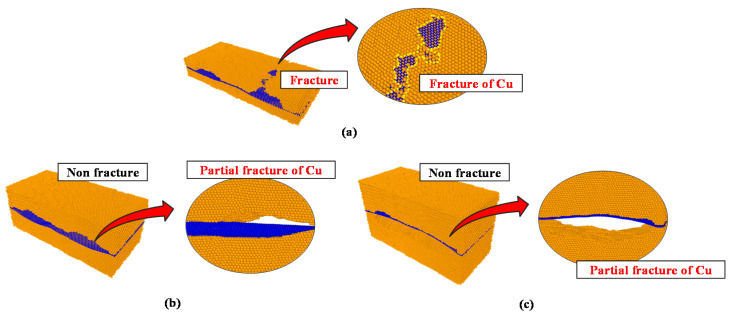
Fracture phenomenon with respect to the zigzag direction. (**a**) Model A; (**b**) model B; (**c**) model C.

However, the fracture strength was only higher than the strength at delamination initiation in the zigzag direction of model A, as shown by the tensile analysis result. The reason for this result is that Cu completely fractured before graphene completely fractured following interface delamination, as shown in Figure 5a.

For model B, the bond of the graphene sheet was broken before Cu completely fractured after delamination initiation. According to Figure 5b, the Cu layer did not fracture completely since the Cu of model B was thicker than the Cu of model A. The remaining Cu layers supported the load, while the hexagonal arrangement of the graphene sheet was broken and fractured first.

Model C, with the thickest Cu layer, exhibited a behavior similar to that of model B, as shown in Figure 5c. The bond of the graphene layer broke just before the Cu layer completely fractured. Therefore, the fracture stress decreased, although delamination initiation began. These results prove that precise analyses are needed to obtain the proper Cu thickness in graphene/Cu composites.

#### 3.1.2. Results of the Armchair-Direction Tensile Analysis

**Figure 6:**
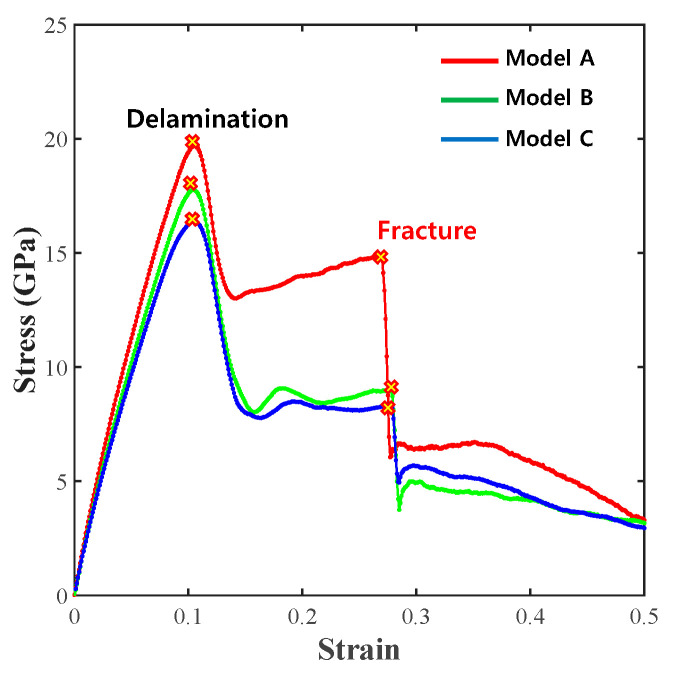
Armchair direction σ–ε results for models A, B, and C.

Model C, with the thickest Cu layer, exhibited a similar behavior to model B, as shown in Figure 6.

The fracture stress of model C was lower than that of model A because the bond of the graphene layer broke just before the Cu layer completely fractured. Therefore, the fracture stress decreased, although delamination initiation began. These results show that precise analyses are needed to obtain the proper Cu thickness in graphene/Cu composites. The results of the analytical model with the tensile force applied in the armchair direction are shown in Figure 6.

The first delamination event occurred when the strain reached approximately 0.1 in models A, B and C, and the strain at the time of fracture was 0.4. Model C had the lowest stress value among the three models at the time of delamination initiation, with values of 16.37 GPa for model C, 17.76 GPa for model B and 19.69 GPa for model A. The stress at the time of delamination initiation decreased as the thickness increased. Additionally, the fracture stresses of models A, B, and C were 14.7, 8.9 and 8.2 GPa, respectively, with the stress decreasing with increasing thickness.

**Figure 7:**
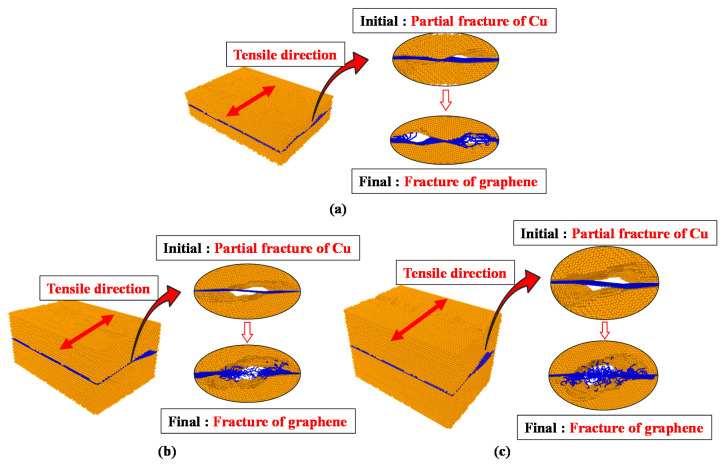
Fracture phenomenon with respect to the armchair direction. (**a**) Model A; (**b**) model B; (**c**) model C.

The overall fracture behavior of models A, B, and C observed in the armchair-direction tensile analysis showed that the entire graphene/Cu composite fractured after interfacial delamination initiation occurred, as observed in the zigzag-direction tensile analysis. A detailed description of the mechanical behavior results obtained for each of these models is shown in Figure 7.

The results of the armchair-direction tensile analysis demonstrate that the bond of the graphene sheet was broken before the Cu layer completely fractured at the point of fracture after delamination initiation, even in model A. Similar behavior was observed in models B and C. In addition, when comparing the stress, the stress at the time of delamination initiation and the fracture stress in the armchair direction according to the tensile analysis results of models A, B and C decreased as the thickness increased. Thus, when the tensile force was applied in the armchair direction, the stress decreased as the Cu layer thickness increased. These results prove that accurate analysis is required to obtain an adequate Cu thickness considering the graphene bond in graphene/Cu composites.

The stress values at the time of delamination and the fracture stress in the armchair direction and the zigzag direction are shown in Table 2.

The fracture stress decreases more rapidly in the armchair direction than in the zigzag direction after delamination; therefore, there is more of a risk of fracture in the armchair direction than in the zigzag direction for graphene/Cu composites.

#### 3.1.3. Results of the Z-Direction Tensile Analysis

**Figure 8:**
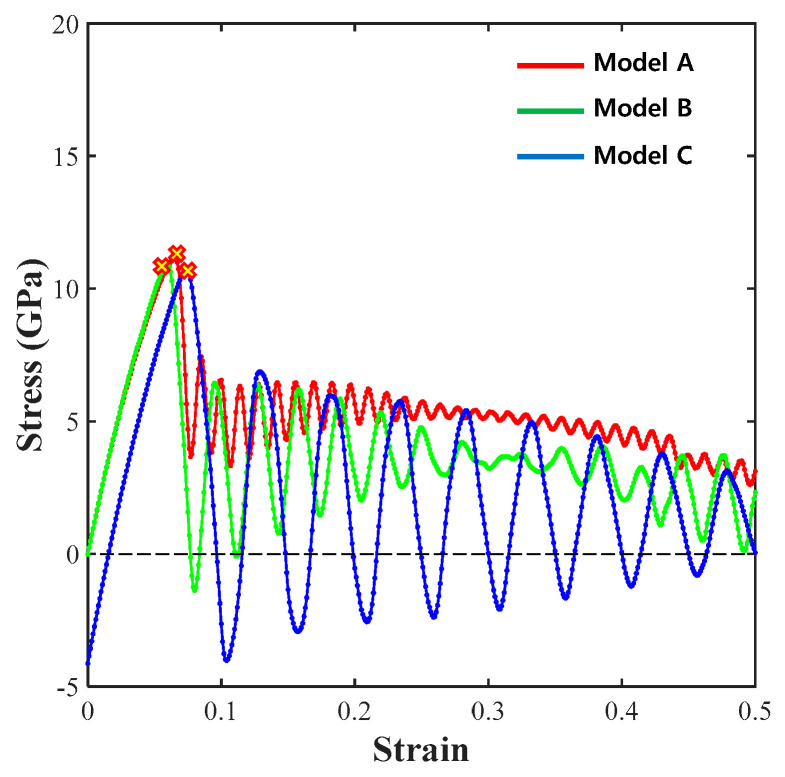
Z-direction σ–ε results for models A, B, and C.

A z-direction tensile analysis was performed to intuitively determine the delamination behavior of graphene and Cu. The stress values of 11.1 GPa for model A, 10.9 GPa for model B, and 10.7 GPa for model C are shown in Figure 8.

Unlike the results of the tensile analyses in the zigzag and armchair directions, the stress value at fracture was unknown because graphene and Cu were completely detached following the initiation of delamination. In addition, since the differences between the increase in the Cu thickness and the stress values at the time of delamination are very small, it is assumed that there is no significant correlation.

### 3.2. Results of the RVE Analysis

In the RVE analysis of the graphene/Cu composites, six cases were analyzed to determine the behavior of the materials and their interfaces and to calculate the macroscopic properties according to the direction in which deformation occurred in the model. For each analysis case, stress variations in the graphene/Cu composites along the x-, y- and z-axes were evaluated from the center of graphene, and graphene was separately evaluated to confirm the intraparticle stress variations.

#### 3.2.1. Results of the $\varepsilon_{\mathrm{xx}}$-Direction Tensile Analysis

**Figure 9:**
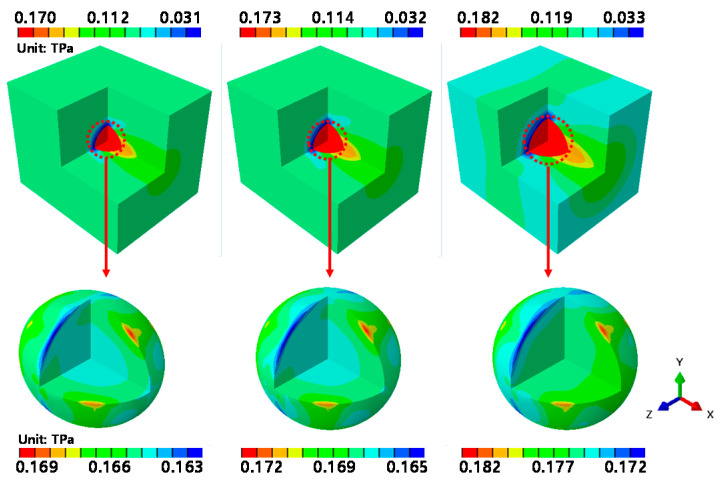
$\varepsilon_{\mathrm{xx}}$-direction RVE analysis results.

Figure 9 shows the stress contours obtained by applying a tensile force to the RVE model of the graphene/Cu composites in the x direction.

From the left, the volume fraction of graphene increases from 1.39% to 2.04% and then to 4.16%. A pattern in the overall stress variations is observed; graphene is subjected to high stress in all analytical models. In the case of the graphene/Cu composites, graphene exhibits high stress in response to external forces. For all of the analytical models, a maximum stress value of 170 GPa is observed when the volume fraction of graphene is 1.39%. When the volume fraction of graphene is 2.04%, the maximum stress is 173 GPa, and when the volume fraction of graphene is at its largest (at 4.16%), the maximum stress increases to 182 GPa.

Additionally, the stress levels and range in Cu increased as the volume fraction of graphene increased. Since all three analytical models had the same applied strain magnitude, this result means that as the volume fraction of graphene under the same loading conditions increases, the stress level in the graphene/Cu composites also increases.

The intraparticle stress variations in graphene are illustrated at the bottom of Figure 9. When a tensile force was applied along the x-axis, the highest stresses appeared in the graphene at the top and bottom of the diagonal of the x-axis, and a low stress variation was observed along the rotational direction of the x-axis. These results indicate that the tensile force applied to the x-axis of the graphene/Cu composites causes greater deformation along the diagonal of the load direction than at the center of graphene.

#### 3.2.2. Results of the $\varepsilon_{\mathrm{yy}}$-Direction Tensile Analysis

**Figure 10:**
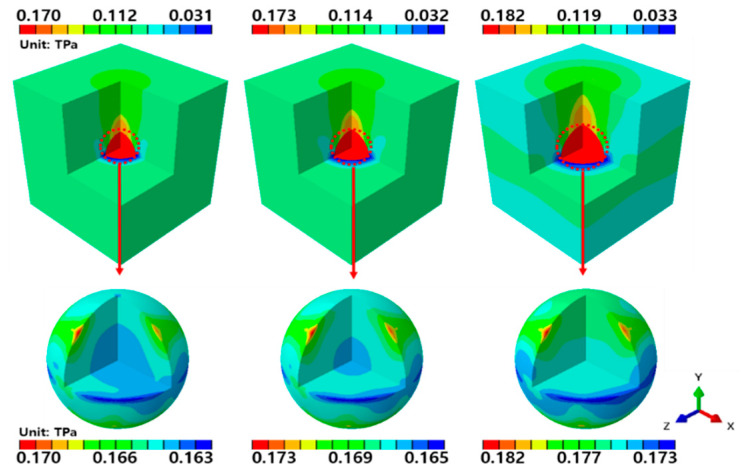
$\varepsilon_{\mathrm{yy}}$-direction RVE analysis results.

Figure 10 shows the stress contours obtained by applying a tensile force to the RVE model of the graphene/Cu composites in the y direction.

From the left, the volume fraction of graphene increases from 1.39% to 2.04% and then to 4.16%. The overall stress variations are similar to those of the x-axis analysis. That is, graphene is under higher stress than Cu, and the stress level increases as the volume fraction of graphene increases.For all of the analytical models, a maximum stress of 170 GPa is observed when the volume fraction of graphene is 1.39%. When the volume fraction of graphene is 2.04%, the maximum stress is 173 GPa, and when the volume fraction of graphene is at its largest (at 4.16%), the maximum stress increases to 182 GPa. The intraparticle stress variations in graphene are similar to those of the x-axis analysis. When a tensile force was applied along the y-axis, the highest stresses appeared in the graphene at the top and bottom of the diagonal of the y-axis, and a low stress variation was observed along the rotational direction of the y-axis. These results indicate that the tensile force applied along the y-axis of the graphene/Cu composites causes greater deformation along the diagonal of the load direction than at the center of the graphene.

#### 3.2.3. Results of the $\varepsilon_{\mathrm{zz}}$-Direction Tensile Analysis

**Figure 11:**
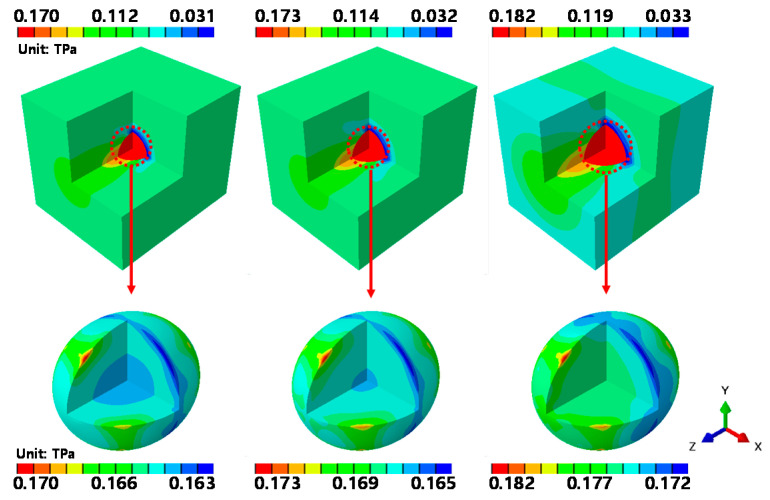
$\varepsilon_{\mathrm{zz}}$-direction RVE analysis results.

Figure 11 shows the contour stresses obtained by applying a tensile force to the RVE model of the graphene/Cu composites along the z-axis, and results similar to those of the previous x- and y-axis analyses are obtained.

For all of the analytical models, a maximum stress of 170 GPa is observed when the volume fraction of graphene is 1.39%. When the volume fraction of graphene is 2.04%, the maximum stress is 173 GPa, and when the volume fraction of graphene is the largest (at 4.16%), the maximum stress increases to 182 GPa. These results are the same as those observed in the y-axis tensile analysis. The stress variations in graphene were also found to be the same as those observed in the x-axis and y-axis tensile analyses. The highest stresses appeared in the graphene at the top and bottom of the diagonal of the z-axis, and a low stress variation was observed along the rotational direction of the z-axis. These results indicate that the tensile force applied along the z-axis of the graphene/Cu composites causes greater deformation along the diagonal of the load direction than at the center of graphene. The same results were also found for the $\varepsilon_{\mathrm{xy}},{\;\varepsilon }_{\mathrm{xz}}{\;\mathrm{and}\;\varepsilon }_{\mathrm{yz}}$ directions analyzed using perturbation analysis.

#### 3.2.4. Stress Variations of the Graphene/Cu Interface

**Figure 12:**
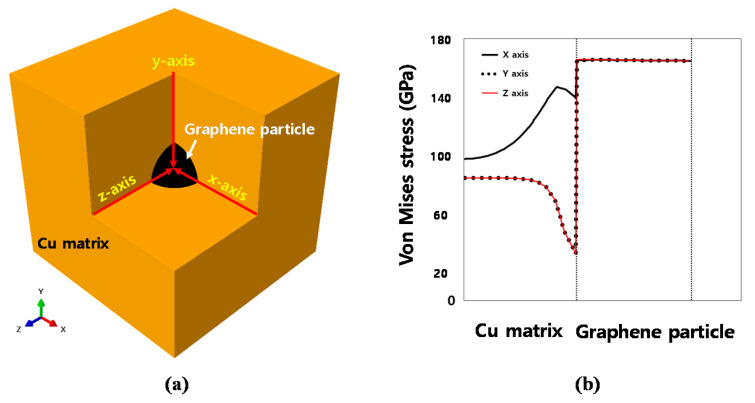
Macroscopic stress variations in the graphene/Cu composites: (**a**) directions; (**b**) Von Mises stress.

Figure 12a shows the stress variations along the x-, y-, and z-axes, from Cu to the center of graphene, for the RVE analytical model in which the volume fraction of graphene was 1.39%.

As with previous results, increasing the volume fraction of graphene only increases the stress value, and all other results show the same trend. Therefore, representatively, the $\varepsilon_{\mathrm{xx}}$ results of the analytical model in which the volume fraction of graphene was 1.39% are shown. In the graph, the first dotted boundary along the horizontal axis indicates the interface between Cu and graphene. The stress along the x-axis, which is the direction of deformation, increases (up to 146 GPa) as the thickness of Cu increases. The stress then begins to decrease near the interface and reaches a minimum value of 139 GPa. Since the maximum stress at the interface of graphene increases instantaneously to 165 GPa, the difference in stress at the interface is 26 GPa. However, the stress along the y- and z-axes shows behavior different from that along the x-axis.

As shown in Figure 12b, a stress level of 84 GPa is maintained for a certain period, regardless of the thickness of Cu. The stress then significantly decreases to 32 GPa at the interface between Cu and graphene. Since the stress at the graphene interface increases to 165 GPa, similar to that observed in the x-axis analysis, the change in stress at the interface with respect to the y-axis and z-axis is very large at 133 GPa. The same phenomenon is observed in the${\;\varepsilon }_{\mathrm{yy}},{\;\varepsilon }_{\mathrm{zz}},{\;\varepsilon }_{\mathrm{xz}}{\;\mathrm{and}\;\varepsilon }_{\mathrm{yz}}$ analyses; the stress difference at the interface in all directions excluding the deformation direction is very large. The large difference between the stresses of the two interfaces implies that the adhesion of the two interfaces is unstable. This means that the graphene/Cu composites may have weaknesses along the interface that do not correspond to the direction in which the external force is applied.

#### 3.2.5. Variation in the Macroscopic Properties of the Graphene/Cu Composites According to the Graphene Size

Equations (2)–(4) (Section 2.1) were applied to $\varepsilon_{\mathrm{yy}},{\;\varepsilon }_{\mathrm{zz}},{\;\varepsilon }_{\mathrm{xy}},{\;\varepsilon }_{\mathrm{xz}}{\;\mathrm{and}\;\varepsilon }_{\mathrm{yz}}$ to calculate the macroscopic constitutive tensor component $\bar{C}$. Since the constitutive tensor is a relational expression of the basic properties of materials, such as the elastic modulus, Poisson’s ratio, and shear modulus, macroscale material properties can be derived. Table 3 shows the macroscale material properties of the RVE calculated in this study according to the graphene volume fraction.

It was calculated that the RVE model with a 1.39% volume fraction of graphene had an elastic modulus of approximately 112 GPa and a Poisson’s ratio of 0.34. This result is attributed to an increase of only 2 GPa in the elastic modulus of Cu. However, when the volume fraction of graphene increased to 4.16%, the elastic modulus increased to approximately 118 GPa, and the Poisson’s ratio decreased to 0.33.

These results imply that an increase in the volume fraction of graphene increases the elastic modulus of the Cu/graphene composites and decreases the Poisson’s ratio. The physical properties calculated and described above are properties that consider the microscopic constituents of the composite material containing graphene, the volume fraction, and the mixed structure of the constituent materials. These properties can be used for a macroscopic structural analysis.

## 4. Conclusions

In this study, uniaxial tensile analyses were conducted considering three different directions to estimate the effects of Cu thickness on structural fracture and stress behavior in graphene/Cu composites. The following conclusions were obtained.

- In the MD analysis results for graphene/Cu composites, the strength performance of the graphene/Cu composites is influenced by whether the graphene maintains its bonds. The strength of the graphene/Cu composites does not increase if the Cu thickness increases. Rather, an increase in Cu thickness in the same tensile direction reduces the strength performance of the graphene/Cu composites.
- Additionally, graphene/Cu composites are more vulnerable to armchair-direction tensile forces than zigzag-direction tensile forces. In graphene/Cu composites, it is necessary to consider the direction of graphene to prevent sudden fracture after delamination.
- In the RVE analysis results for the graphene/Cu composites, a large stress occurred in graphene, which resulted in a large stress difference between graphene and Cu. This means that the interface of graphene/Cu composites is most vulnerable to external loads. The difference according to the volume fraction change was that when the volume fraction of graphene increased, the stress distribution of Cu also increased.
- In the RVE analysis results for graphene where the stress is most concentrated, even within graphene, various stress distributions were observed. These results disprove the necessity of RVE multiscale analysis for graphene/Cu composites. In this study, it was found that higher stress appeared at the interface than at the center of graphene.
- Further work should be performed with the aim of evaluating graphene/Cu composites that laminated multi-layer graphene rather than single-layer graphene. This further work will validate the usage specifications and performance of graphene/Cu composites in practical applications such as semiconductor wiring and electronic devices.

## Figures and Tables

**Table 1:** Properties of Cu and graphene in the RVE model.

Material	Young’s Modulus (Elastic Modulus)	Poisson’s Ratio
Cu	110 GPa	0.34
Graphene	1 TPa	0.149

**Table 2:** The stress values at the time of delamination and the fracture stress in the armchair direction and the zigzag direction.

Model	Stress (GPa)			
	Zigzag Direction		Armchair Direction	
	Delamination	Fracture	Delamination	Fracture
**Model A**	15.53	19.7	16.37	14.7
**Model B**	17.02	11.5	17.76	8.9
**Model C**	16.03	8.9	19.69	8.2

**Table 3:** The macroscale material properties of the RVE results.

**Graphene: 1.39%**		
E1 (GPa)	E2 (GPa)	E3 (GPa)
112.654	112.655	112.655
Nu12	Nu13	Nu23
0.34	0.34	0.34
G12 (GPa)	G13 (GPa)	G23 (GPa)
42.049	42.049	42.049
**Graphene: 2.04%**		
E1 (GPa)	E2 (GPa)	E3 (GPa)
113.951	113.953	113.952
Nu12	Nu13	Nu23
0.34	0.34	0.34
G12 (GPa)	G13 (GPa)	G23 (GPa)
42.528	42.528	42.519
**Graphene: 4.16%**		
E1 (GPa)	E2 (GPa)	E3 (GPa)
118.422	118.428	118.427
Nu12	Nu13	Nu23
0.33	0.33	0.33
G12 (GPa)	G13 (GPa)	G23 (GPa)
44.050	44.049	44.051

## Data Availability

The data presented in this study are available on request from the corresponding author.
